# An effective technique for preventing the subcutaneous migration of the abdominal lumboperitoneal shunts catheters

**DOI:** 10.1186/2045-8118-12-S1-P22

**Published:** 2015-09-18

**Authors:** Takashi Kawahara, Masamichi Atsuchi, Hiroshi Tokimura, Tetsuzo Tomosugi, Kazuho Hirahara, Kazunori Arita

**Affiliations:** 1 Department of Neurosurgery, Kgoshima City Hospital, Kagoshima, Japan; 2 Division of Neurosurgery, Atsuchi Neurosurgical Hospital, Kagoshima, Japan; 3 Department of Neurosurgery, Graduate School of Medical and Dental Sciences, Kagoshima University, Kagoshima, Japan

## Introduction

Migration of the lumboperitoneal shunt catheter into the abdominal subcutaneous space is not uncommon. We devised a new simple method (Transrectus Gap method we call) for installment of peritoneal tube aiming to prevent the migration.

## Methods

After catheter insertion into the lumber spinal subarachnoid space peritoneal side tube was drawn into areola vertical space between abdominal fat and superficial fascia of rectus muscle. After a 4 cm incision of on the superficial rectal fascia and split the rectus muscles, the tip of catheter was obliquely passed through abdominal rectus muscle using mosquito clamp. The tube was then inserted into abdominal cavity through a small hole on the deep fascia and peritoneal membrane which was 3 cm down to the hole on anterior rectal sheath.

## Results

Thus, the peritoneal side catheter ran obliquely, upper lateral to lower medial, through anterior sheath, abdominal rectus muscle, and inserted to the peritoneum. We have so far operated 120 patients with this method without major complication or migration of the catheter.

## Conclusion

This technique installs the abdominal catheter run parallel to the abdominal wall. As the result, the influence of the abdominal pressure to the abdominal catheter seems reduced. And the catheter does not pass the dead space made by operation, it is another reason preventing the subcutaneous migration.

## References

[B1] Use of blunt scalp hooks for abdominal procedure in lumboperitoneal shunt placement: technical note Neurol Med Chir (Tokyo) 2014 54 7 552 3 Epub 2014 Apr 23 10.2176/nmc.tn.2013-0379 24759096 PMC4533468

[B2] Surgical technique for preventing subcutaneous migration of distal lumboperitoneal shunt catheters Innovative Neurosurgery 2013 1 3-4

